# Systematic review of robotic liver resection

**DOI:** 10.1007/s00464-012-2547-2

**Published:** 2012-12-12

**Authors:** Cheng-Maw Ho, Go Wakabayashi, Hiroyuki Nitta, Naoko Ito, Yasushi Hasegawa, Takeshi Takahara

**Affiliations:** 1 Department of Surgery, Iwate Medical University School of Medicine, 19-1 Uchimaru, Morioka, Iwate 020-8505 Japan; 2 Department of Surgery, National Taiwan University Hospital, Taipei, Taiwan

**Keywords:** Robotic liver resection, Systematic review, Minimally invasive surgery

## Abstract

**Background:**

Robotic liver resection has emerged as a new modality in the field of minimally invasive surgery. However, the effectiveness of this approach for liver resection is not yet known.

**Methods:**

A literature survey was performed using specific search phrases in PubMed. Case series that focused on biliary reconstruction were excluded. Characteristics, such as patient demographics, perioperative outcomes, and oncological results for colorectal liver metastasis and hepatocellular carcinoma were analyzed.

**Results:**

Nineteen series that described the cases of 217 eligible patients were reviewed. The most commonly performed procedures were wedge resection and segmentectomy. Right hepatectomy was performed in a few specialized centers. The conversion and complication rates were 4.6 and 20.3 %, respectively. The most common reason for conversion was unclear tumor margin. Intra-abdominal fluid collection was the most frequently occurring morbidity. Mean operation time was 200–507 min. Mean intraoperative blood loss was 50–660 mL, with a tendency toward increased blood loss observed in series that included major hepatectomies. Mean postoperative hospital stay was 5.5–11.7 days. The longest mean follow-up time was 36 months for colorectal liver metastasis and 25.1 months in hepatocellular carcinoma. Disease-free survival for mixed malignancies was comparable to that after laparoscopic procedures. Overall survival was not reported.

**Conclusions:**

Robotic liver resection is safe and feasible for experienced surgeons with advanced laparoscopic skills. Long-term oncologic outcomes are unclear, but short-term perioperative results seem comparable to those of conventional laparoscopic liver resection.

In the 1990s, liver resection was known to be associated with high morbidity and mortality because of the complexity of the vascular and biliary structures of the liver, exposure difficulties, and propensity for bleeding during manipulation. With the advancement of surgical techniques, development of instruments for regulating hemostasis, and improvement of postoperative care, the success rate of liver resection surgeries has improved significantly along with the oncological outcomes for patients with hepatocellular carcinoma (HCC) [1]. The emergence of minimally invasive surgery for liver resection procedures has thrived with the introduction of novel technologies, including flexible fiberoptic imaging systems, and hemostatic options, such as clips, staplers, and electrical or ultrasonic energy-induced hemostasis, and laparoscopic liver resection, has been shown to be safe in experienced hands, with acceptable morbidity and mortality rates for both minor and major hepatic resections [2, 3]. Previous studies conducted on selected groups of patients have shown that the 5-year survival rates for patients undergoing laparoscopic HCC resection were comparable to those of patients undergoing open hepatic resection [2, 4]. The advantages of minimally invasive surgery are well known. Shorter hospital stays, decreased postoperative pain, rapid return to preoperative activity, improved cosmesis, and decreased postoperative ileus are among the benefits of the laparoscopic approach [3]. However, laparoscopic liver surgery, although it has benefitted from advances in minimally invasive surgery, is not without inherent limitations, including limited degrees of freedom for manipulation, fulcrum effect against the port, tremor amplification, awkward ergonomics, and two-dimensional imaging adaptation [5].

Himpens et al. [6] reported the first successful clinical application of telerobotics in 1997, when they performed a laparoscopic cholecystectomy using a da Vinci prototype. Robotic surgery features EndoWrist instruments, providing 7 degrees of freedom for instrument movement and tremor filtering. It allows surgeons to be in a seated posture for long operation tolerance and permits three-dimensional imaging, real-time radiographic correlation, and easy suture maneuvering [5, 7]. Various general surgical procedures have been performed using surgical robots, including cholecystectomy, Nissen fundoplication, Heller myotomy, Roux-en-Y gastric bypass, and, more recently, colorectal surgery [8, 9]. Hyung concluded that the application of robotic technology for general surgery is technically feasible and safe, improving dexterity, allowing for better visualization, and attaining a high level of precision [8]. However, the absence of tactile sensations and the extremely high costs of such technologies are still major problems to be solved [8].

We hypothesized that the advantages of robotic surgical technology could translate to and be effectively applied in liver resection. The purpose of this study was to evaluate critically the reported cases of robotic liver resection and to analyze the surgical and oncologic outcomes.

## Materials and methods

### Literature review of published robotic liver surgeries focused specifically on resections

A literature search was performed using the PubMed database with the search phrases “robotic liver surgery,” “robotic liver resection,” “robot hepatic surgery,” “robotic hepatic resection,” or “robotic liver.” All titles and abstracts were screened for review, with careful examination of the data to remove double counting of patients between series. Series focused on biliary reconstruction (choledochal cyst or biliary atresia) were excluded. Patient demographics (age, sex, and indication for surgery), perioperative characteristics (operating maneuvers), outcomes (operation time, blood loss, transfusion requirement, conversion, complications, and hospital stay), and documented oncological outcomes for colorectal liver metastasis (CRLM) and HCC (tumor size, recurrence, and survival) were analyzed.

## Results

### Search results and baseline characteristics of patients in the included studies

**Fig. 1:**
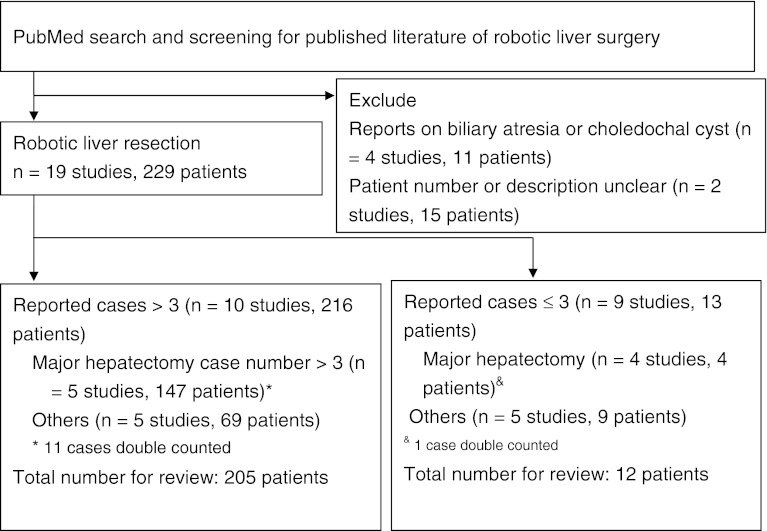
Systematic review of the robotic liver resection flow diagram (217 patients)

A total of 25 publications, including 255 patients, were relevant to robotic liver surgery. Of these, 19 publications (229 patients) that focused on liver resection and provided specific patient descriptions were included in this review [10–28]. After removing doubly counted cases, 217 patients were eligible for inclusion within this study (Fig. 1). Ten studies were large case series [10–19], two of which also were comparative studies using conventional laparoscopic surgery or open surgery [14, 16]. Each of these ten studies included more than three patients, accounting for the majority of cases (207 patients, 95.4 % of total reported cases). Two case series had three patients each [20, 21], and there were seven single-case reports [22–28]. The baseline characteristics of patients within the included studies are listed in Table 1. The number of patients in each study ranged from 1 to 70. All studies used the da Vinci robot system (Intuitive, Sunnyvale, CA).

**Table 1:** Publications of robotic liver resection (listed by number of patients)

Authors	Years	Journal	No. patients	Malignant	Benign
Giulianotti et al.^a^	2011	Surgery	70	42	28
Choi et al.	2012	Surg Endosc	30	21	9
Chan et al.	2011	J Hepatobiliary Pancreat Sci	27	21	6
Giulianotti et al.	2011	Arch Surg	24	17	7
Casciola et al.	2011	Surg Endosc	23	19	4
Ji et al.	2011	Ann Surg	13	8	5
Lai et al.	2012	Int J Surg	10	9	1
Berber et al.	2010	HPB	9	9	0
Patriti et al.	2009	J Hepatobiliary Pancreat Surg	6	6	0
Wakabayashi et al.	2011	J Hepatobiliary Pancreat Sci	4	3	1
Choi et al.	2008	Yonsei Med J	3	2	1
Vasile et al.	2008	Chirurgia (Bucur)	3	1	2
Panaro et al.	2011	JSLS	1	1	0
Holloway et al.	2011	Gynecol Oncol	1	1	0
Giulianotti et al.	2010	J Laparoendosc Adv Surg Tech A	1	1	0
Machado et al.	2009	Arq Gastroenterol	1	1	0
Giulianotti et al.^b^	2011	J Hepatobiliary Pancreat Sci	1	0	1
Giulianotti et al.	2012	Transplant Int	1	0	1
Ryska et al.	2006	Rozhl Chir	1	0	1

^a^Excluded minor procedures (wedge resections, biopsies, enucleation, and simple liver cyst fenestration)

^b^Included in Giulianotti et al., Arch Surg 2011

### Indications for robotic liver resection

The indications for robotic hepatic resection included benign liver lesions and malignancy (Table 2). The upper limit of tumor size was 5–6 cm in most series, whereas Giulianotti et al. [10] did not report a size limitation. One live-donor transplantation of the right lobe of the liver also was performed [27]. The contraindications to robotic liver resection include any of the contraindications for open liver surgery along with pneumoperitoneum intolerance and presence of extensive lesions that have invaded major vascular structures or require vascular reconstruction.

**Table 2:** Indications and contraindications for robotic liver resection

Indications	Contraindications
Benign liver lesions	Any contraindications to open liver resection (cardiac or respiratory insufficiency, or ASA status > 3)
Symptomatic hemangioma	Pneumoperitoneum intolerance
Symptomatic FNH	Lesions with extensive subcapsular involvement
Adenoma	Lesions invading major hepatic vessels^c^
Biliary hamartoma Schwannoma	The need for vascular reconstruction
Hepatolithiasis	
Cystic lesions^a^	
Recurrent pyogenic cholangitis	
Malignant liver lesions	
Tumor size < 6 cm	
HCC	
Cholangiocarcinoma	
CRC metastasis^b^	
Other malignant lesions	
Live donor hepatectomy for liver transplant	
Indeterminate lesions	

*ASA* American Society of Anesthesiologists, *FNH* focal nodular hyperplasia, *HCC* hepatocellular carcinoma, *CRC* colorectal carcinoma

^a^Including symptomatic giant hepatic cysts and hydatid cysts

^b^In the absence of peritoneal carcinomatosis or unresectable extrahepatic disease

^c^Including portal vein branches, the inferior vena cava, and major hepatic veins

### Types of robotic liver resections

The most commonly reported procedure for robotic liver resection was wedge resection or segmentectomy (37.7 %), followed by right hepatectomy (21.6 %), and left lateral segmentectomy (20.8 %; Table 3). Most of the reported cases of right hepatectomy (33/51) were contributed by a single surgeon (Giulianotti et al. [10, 12, 26, 27]).

**Table 3:** Types of robotic liver resections performed in the literature reviewed

Total reported procedures	236
Wedge resection/segmentectomy	87 (37.7 %)
Left lateral sectionectomy	51 (20.8 %)
Left hepatectomy^a^	31 (13.1 %)
Bisegmentectomy	12 (5.1 %)
Right hepatectomy	51 (21.6 %)
Right trisectionectomy	2 (0.8 %)
Right live donor hepatectomy	1 (0.4 %)
Extended right hepatectomy^b^	1 (0.4 %)
Pericystectomy	2 (0.8 %)

^a^Included one case of caudate segmentectomy and one case of Roux-en-Y hepaticojejunostomy

^b^With Roux-en-Y hepaticojejunostomy

### Surgical technique

The port setting in the robotic technique is a little different from the conventional laparoscopic setting. Five or six ports were used (3 for the robotic working arm, 1 for the robotic camera, and 1 or 2 for the assistant working port). The camera port was usually placed in the umbilical or right paraumbilical area, or, in the case of the Berber et al. series, 20 cm away from the tumor and 10 cm from the working robotic port. The umbilical port was reserved, in the latter case, for the assistant to perform retraction, clipping, stapling, and suction. The fourth robotic arm was generally used for lobe exposure or tenting to create a new working space for dissection of the inferior vena cava [10]. Parenchymal transection was performed using a robotic harmonic device or robotic bipolar electrocautery with Maryland forceps for crushing with or without precoagulation treatment. In the series reported by Chan et al., the assistant used an ultrasonic aspiration dissector for fine parenchymal dissection (Cavitron Ultrasonic Surgical Aspirator, CUSA, Valleylab Inc., Boulder, CO) [11]. Vascular and biliary elements were divided by the assistant using a harmonic scalpel, clips, scissors, or stapler, when appropriate. Hemostasis of small vessels was performed with monopolar or bipolar cautery, whereas larger vessels were secured with clips, ligature, or running suture, the last of which is considered to provide a considerable advantage over conventional laparoscopic liver resection.

### Conversion and complications

Conversion was reported in ten (4.6 %) cases: nine to laparotomy and one to a hand-port laparoscopic procedure (Table 4). The reasons for conversion included doubling of the tumor margin, bleeding control, long resection plane, anatomical distortion of hilum due to severe adhesion, and obesity. No cases of surgical mortality were reported. The reported morbidity was 20.3 % (48/236). The most common complication was intra-abdominal collection of bile or abscess (Table 4). Transient liver failure and deep vein thrombosis were reported at a higher frequency. Portal vein stenosis was noted in one donor 6 months after robotic living donor right hepatectomy [27]. The authors in this case suggested that the mechanism was either angulation of the portal vein caused by hepatic regeneration or formation of a band of scar tissue; intraoperative injury was not suggested based on the normal CT angiogram at 1 month after the operation. The patient had a 70 % stenosis of the main trunk of the portal vein and required percutaneous transhepatic dilatation. The patient recovered well during the 1-year follow-up period.

**Table 4:** Reasons for conversion from robotic to open surgery and reported complications

Conversion	Case number
To open laparotomy	
Unclear tumor limits/margin	3
Bleeding	3
Anatomical distortion of hilum due to severe adhesion	1
Long resection plane	1
Obesity	1
To hand-port laparoscopic surgery	
Bleeding	1
Complications	48
Intra-abdominal collection/bile leak/abscess	16
Intraoperative bleeding requiring transfusion	4
Transitory liver failure	3
Deep vein thrombosis	3
Wound infection	3
Incisional hernia	2
Reoperation^a^	2
Pleural effusion	2
Transient ischemic cerebral attack	2
Postoperative bleeding	1
Urinary bladder injury	1
Portal vein stenosis	1
Prolonged trocar-site pain	1
Prolonged ascites	1
Colonic anastomotic failure	1
Empyema	1
Pneumonia	1
Prolonged ileus	1
Hepatitis B viral reactivation	1
Hepatic venous congestion	1

^a^Due to concomitant colon anastomotic failure

### Patient demographics and perioperative outcomes

Table 5 lists patient demographics and perioperative outcomes. Mean ages ranged from 52 to 73 years. There were 96 men and 109 women included in this review. Mean operation time ranged from 200 to 507 min. In two comparative studies, Berber et al. [16] found no differences in the mean operation times of the robotic and laparoscopic procedures (*P* = 0.4), whereas a cohort-matched study conducted by Ji et al. [14] suggested that the robotic procedure required longer operation times than laparoscopic and open resection surgeries. Mean intraoperative blood loss ranged from 50 to 660 mL. There was a tendency for patients to experience more blood loss during hepatectomies or combined colorectal surgeries [12, 15, 17]. No difference in blood loss was noted between robotic and laparoscopic surgeries in the series reported by Berber et al., whereas in the study by Ji et al. less blood loss was reported during robotic procedures than during laparoscopic and open resection procedures. The mean postoperative hospital stay ranged from 5.5 to 11.7 days. In the series by Ji et al. [14], the mean postoperative stay was shorter for traditional laparoscopic procedures (5.2 days) than for the robotic procedure (6.7 days) and open surgical procedures (9.6 days), possibly due to restricted patient selection and simpler laparoscopic procedures. However, conversion from traditional laparoscopic to open and hand-assisted laparoscopic resection was necessary in two patients who underwent right hemihepatectomy and left hepatectomy, whereas no conversions occurred in the robotic group [14].

**Table 5:** Patient demographics and perioperative outcomes (reports with more than 2 patients)

Authors	Years	No. patients	Age, yr (range and/or SD)	Male:female ratio	Major hepatectomy (>2 segmentectomy)	Operation time, min (range and/or SD)	Intraoperative blood loss mL (range and/or SD)	Transfusion	Postoperative hospital stay days (range or SD)
Giulianotti et al.	2011	70	60 (21–84)	30:40	27/70	270 (90–660)	262 (20–2,000)	15/70	7 (2–26)
Choi et al.	2012	30	52.4 (28–71)	14:16	20/30	507 (120–812)	343 (95–1,500)	4/30	11.7 (5–46)
Chan et al.	2011	27	61 (37–85)	16:11	1/27	200 (90–307)	50 (5–1,000)	NM	5.5 (3–11)
Giulianotti et al.	2011	24	55 (21–84)	10:14	24/24	337 (65)	457 (100–2,000, 401)	3/44	9.0 (3.0)
Casciola et al.	2011	23	66.4 (32–84, 13.4)	15:8	0/23	280 (101)	245 (254)	NM	8.9 (9.4)^b^
Ji et al.	2011	13	NM	NM	9/13	338	280	0/13	6.7
Lai et al.	2012	10	65.1 (13.8)	5:5	10/10	347.4 (85.9)	407 (286.8)	1/10	6.7 (3.5)
Berber et al.	2010	9	66.6 (6.4)	2:7	0/9	258 (27.9)	136 (61)	NM	NM
Patriti et al.	2009	7	73 (3.7)	3:4	1/7	334 (37)	660 (250.7)^a^	NM	8.1 (0.3)
Wakabayashi et al.	2011	4	NM	NM	0/4	272 (40.4)	Negligible	0/4	NM
Vasile et al.	2008	3	NM (30–58)	1:2	0/3	NM (120–160)	NM (70–150)	0/3	7
Choi et al.	2008	3	64 (2.6)	1:2	0/3	463.3 (76.4)	366.7 (144)	1/3	8.3 (1.9)

*SD* standard deviation, *NM* not mentioned

^a^Included time for robotic colectomy for all cases

^b^One stay was for 46 days for ileocolic anastomotic failure of the concomitant right colectomy

### Oncological outcomes after robotic liver resection for CRLM and HCC

Most series reported mixed results for outcomes in patients with malignancies (Table 6). Reported mean tumor sizes ranged from 1.5 to 6.4 cm. The longest mean follow-up time was 36 months in patients with CRLM and 25.1 months in patients with HCC. No port-site recurrence was reported. Three patients with CRLM and three with HCC had recurrence within 1 year. Berber et al. [16] reported that disease-free survival for mixed malignancies was comparable in patients undergoing robotic and laparoscopic procedures. Overall survival was not reported in any of the included studies.

**Table 6:** Oncologic outcomes after robotic liver resections for CRLM and HCC

Authors	Years	No. patients	Tumor size (cm) (range and/or SD)	Mean follow-up (months) (range and/or SD)	Postoperative oncologic outcome
CRLM					
Giulianotti et al.	2011	16	NM	NM	
Casciola et al.	2011	14	NM	25.1 (11.7)^e^	2 died due to tumor progression
					3 alive with malignant disease (1 lung, 1 lung and nodal, and 1 liver)
Giulianotti et al.	2011	11	5.2 (2.8)	36 (1–57)	2 patients with recurrent CRLM at 10 and 20 months, and patients underwent second liver resections. Both patients are alive and disease-free when the study was published
					1 patient with bilateral pulmonary metastasis receiving chemotherapy and still alive when the study was published
					1 patient died 12 months postoperatively because of cerebral metastasis detected during the ninth month after the operation
Lai et al.^a^	2012	7	3.8 (1–6, 1.6)	Less than 1 year	1 patient used RFA to manage bilobar lesions
Patriti et al.	2009	6	NM	6.3 (1–11)	1 recurrent CRLM at 7 months
Choi et al.	2012	4	NM	12 (3–22)	1 recurrent CRLM at 5 months
Berber et al.^b^	2010	4	3.2 (1.3)	14^f^	1 recurrent CRLM
Choi et al.	2008	1	1.5	NM	
HCC					
Choi et al.	2012	13	3.1 (0.8–5)	12.2 (5–23)	Alive and no recurrence
Giulianotti et al.	2011	13	NM	NM	
Ji et al.^c^	2011	6	6.4 (1.8–12)	NM	
Casciola et al.	2011	3		25.1 (11.7)^e^	1 patient died due to tumor progression
Berber et al.	2010	3		14^f^	1 local recurrence 6 months after resection
Lai et al.^a^	2012	2	3.8 (1–6, 1.6)	Less than 1 year	1 local recurrence
Panaro et al.	2011	1		4	
Giulianotti et al.^d^	2011	1		6	Alive and no recurrence
Machado et al.	2009	1		4	
Choi et al.	2008	1		3	1 hepatic recurrence and portal vein thrombi at 3 months

*CRLM* colorectal liver metastasis, *HCC* hepatocellular carcinoma, *NM* not mentioned

^a^Mixed results for all 9 patients with malignancy

^b^Disease-free survival of the mixed results was equivalent to the laparoscopic group

^c^Mixed results for all 13 patients

^d^Mixed results for all 24 patients

^e^Mixed results for all 19 patients with malignancy

^f^Mixed results for all 9 patients with malignancy

## Discussion

Robotic liver resection is emerging as a new minimally invasive surgical technique incorporating conventional laparoscopic procedures with a patient-side surgeon and remote robotic control of instruments by a console surgeon. This review included reports of more than 200 patients who underwent robotic liver resections. The reported rates of conversion and complications, although possibly underestimated because of selection and publication biases, were acceptable at 4.6 and 20.3 %, respectively. Of the two comparative studies, the rate of complications reported during robotic liver resection was comparable to that of conventional laparoscopic procedures [14, 16]. Most series concluded that robotic liver resection was safe and feasible when performed by experienced surgeons. In fact, there is even a report of a case of a laparoscopic wedge resection of liver segments 7 and 8 that was “converted” to robotic-assisted surgery because of an Endo GIA stapler malfunction [28]. In this case, Boggi et al. [29] demonstrated the usefulness of robotic suture in large caval injuries. Idrees and Bartlett claimed that the features of the da Vinci robot, including the use of three robotic arms by the same operating surgeon, use of articulating instruments that can be locked in place as vascular clamps, and ability to perform intracorporeal suturing and tying in difficult locations, are extremely useful in controlling and definitively managing bleeding without necessitating an open surgery [7]. They also noted that the ability to lock the articulating instruments in place as a substitute for vascular clamping could be invaluable, because it gives the anesthesia team time to resuscitate a patient and the surgical team time to formulate a management plan when bleeding complications arise [7]. However, it should be noted that the lack of tactile feedback when performing suture and knot tying with the robotic instruments might lead to uncontrolled tissue overstretching injury or suture disruption (personal communication). Careful visual observation for compensation is mandatory. Otherwise, it is prudent and reasonable to conclude that robotic liver resection is a procedure that can be safely completed by experienced surgeons.

The most commonly performed robotic liver resection procedures were partial resection and segmentectomy, followed by left lateral segmentectomy and right hepatectomy. However, most reported cases of right hepatectomy were performed in highly specialized centers, and these series also reported a tendency toward more intraoperative blood loss [12, 15]. This can be interpreted with caution, because these cases did not represent the current norm in robotic liver resection. In our experience, wristed instruments can improve the looping and isolation of the left Glissonian pedicles, which is very useful in left-sided hepatic resection. On the other hand, right-sided liver resection, which often requires full mobilization and is difficult to achieve without tilting the operating table, is frequently performed using robotic arms that are docked following full mobilization of the liver by laparoscopic instruments and a flexible laparoscopic camera [7]. Wristed instruments also can aid in suturing and knot tying during laparoscopy, which is helpful for vessel control and hemostasis at the bleeding point. Thus, robotic liver resection is feasible for many types of procedures, but the application of robotic techniques to every procedure by all liver surgeons is still considered a challenge.

Unlike other procedures, robotic liver resection requires a team approach that should include a highly skilled laparoscopic surgeon at the patient’s side to manage complex instruments and techniques, such as the harmonic scalpel, clipping, stapling, and even the use of LigaSure or CUSA. The installation and exchange of robotic arms also requires experienced personnel. Adequate training is indispensable to facilitate the use of robotic surgical equipment [30]. Some authors have suggested that mastering the robot requires at least ten robotic procedures in robot-assisted laparoscopic surgery [31]. A cumulative sum analysis has demonstrated the learning curve for laparoscopic hepatectomies over the course of 60 cases [32], but no data are available yet regarding the learning curve for robotic liver resection. It is possible that the learning curve for robotic resections may be shorter than that of conventional laparoscopic liver surgery, because the three-dimensional imaging camera, wristed instruments, and better ergonomics will help already experienced laparoscopic surgeons to quickly familiarize themselves with the robotic procedure.

Our study reviewed cases of robotic liver resection currently reported in the literature. However, some of the series chosen for this study did not clearly describe patient demographics or specific outcomes. Our summary, especially in terms of oncologic outcomes, was not representative of all studies on this topic. Most of the reported series found to date have focused on short-term perioperative outcomes. Long-term results and cost-effectiveness are expected to be reported in future studies and are necessary before the advantages and disadvantages of robotic liver resection can be conclusively stated.

## Conclusions

Robotic liver resection is safe and feasible in experienced hands. It requires an expert patient-side surgeon with advanced laparoscopic skills. Wristed instruments are useful in a variety of maneuvers, such as looping Glissonian pedicles (especially on the left side of the liver) and in suturing bleeding points. Long-term oncologic outcomes are unclear, but short-term perioperative results indicate that robotic liver resection is comparable to conventional laparoscopic liver resection.
